# Intestinal epithelial Tet2 deficiency reprograms the gut microbiota through bile acid metabolic alterations

**DOI:** 10.1128/mbio.03562-25

**Published:** 2026-01-26

**Authors:** Nan Wang, Qing Liu, Fengjiao Huo, Shuaishuai Zhang, Shuyao Lv, Taotao Mi, Hailiang Liu

**Affiliations:** 1 Institute for Regenerative Medicine, State Key Laboratory of Cardiology and Medical Innovation Center, Shanghai East Hospital, School of Medicine, Tongji University 481875, Shanghai, China; 2 Institute of Advanced Biotechnology and School of Medicine, Southern University of Science and Technology 255310, Shenzhen, China; California State University Northridge, Northridge, California, USA

**Keywords:** Tet2, bile acid, ASBT, gut microbiota

## Abstract

Epigenetic mechanisms are increasingly recognized as critical regulators of host–microbiota interactions, yet their specific roles in gut homeostasis remain elusive. Here, we demonstrate that intestinal epithelial-specific deletion of the DNA demethylase Tet2 leads to structural abnormalities, impaired barrier function, and remarkable reprogramming of the gut microbiota. Mechanistically, Tet2 deficiency downregulated the apical sodium-dependent bile acid transporter ASBT/Slc10a2, resulting in altered bile acid homeostasis with luminal accumulation of hyocholic acid (HCA). This metabolic shift created a favorable niche for the selective expansion of bile salt hydrolase (BSH)-expressing Lactobacillus species. Furthermore, we identified an age-dependent regulatory role of HCA, which promoted Lactobacillus in young mice but enriched *Akkermansia* in aged animals. Our findings establish an epigenetic-metabolic-microbial axis centered on Tet2-mediated bile acid regulation, providing new insights into how host epigenetic factors shape the gut microbial ecosystem in an age-sensitive manner.

**IMPORTANCE:**

While the gut microbiota is known to influence host physiology, the molecular mechanisms by which the host epigenetically regulates microbial composition remain largely unexplored. Our work reveals that the epigenetic enzyme Tet2 in intestinal epithelial cells acts as a master regulator of gut microbial ecology by modulating bile acid metabolism. The discovery that Tet2 deletion drives hyocholic acid (HCA) accumulation—which exerts age-dependent effects on *Lactobacillus* and *Akkermansia*—provides a novel principle for understanding host–microbe interactions across the lifespan. By linking epithelial DNA demethylation to bile acid transport and microbial phenotype, we establish a previously unrecognized Tet2-ASBT-HCA pathway that expands the conceptual framework for microbiota research. These insights open new avenues for therapeutic interventions aimed at reversing microbial dysbiosis through epigenetic or metabolic modulation.

## OBSERVATION

Epigenetic mechanisms are increasingly acknowledged as pivotal modulators of host–microbiota interactions. Ten-eleven translocation methylcytosine dioxygenase 2 (Tet2), an enzyme responsible for DNA demethylation, regulates levels of 5-hydroxymethylcytosine (5hmC) and has been associated with aging and neurodegenerative diseases (1). Additionally, evidence suggests that TET2 activity may affect intestinal homeostasis and microbial composition, although this relationship remains inadequately characterized (2–5). In this study, we investigated the role of Tet2 and the bile acid (BA) transporter ASBT in influencing gut microbiota composition. Our findings collectively underscore the interplay between intestinal Tet2 deletion, DNA methylation, BA transport, and microbiota remodeling. To investigate the functional role of TET2 in the gut, we generated intestinal epithelial-specific Tet2 knockout mice (Tet2-iKO) by crossing Tet2^fl/fl^ (WT) mice with Villin-Cre mice (6).

**Fig 1:**
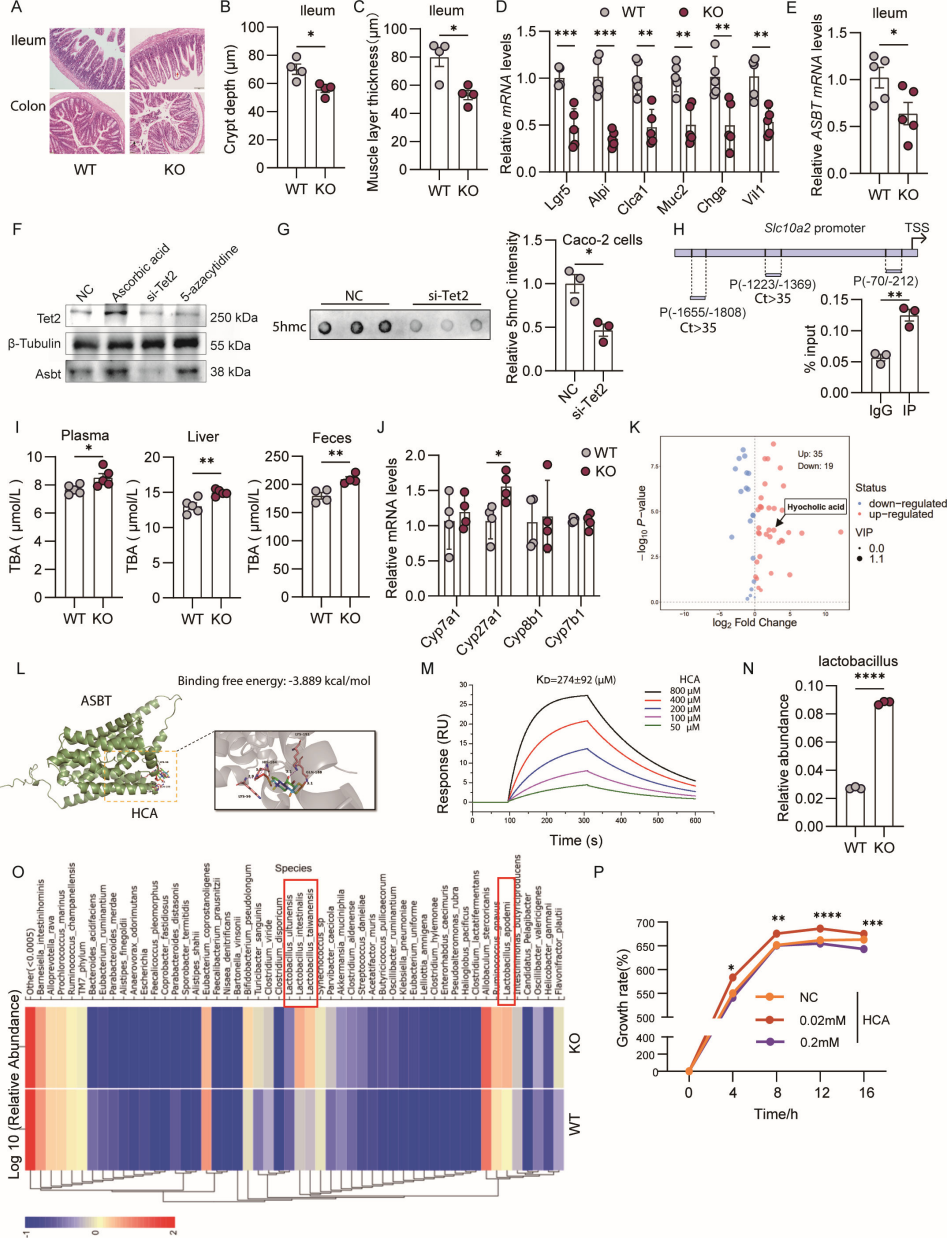
The impact of intestinal epithelial Tet2 deficiency on epithelial architecture, bile acid metabolism, and gut microbiota composition. (**A**) Representative hematoxylin and eosin (H&E) stained images of the ileum and colon are presented. Tet2-iKO mice exhibit submucosal gaps at the villus tips (indicated by red arrows) and focal epithelial shedding with exposed lamina propria in the colon (indicated by black arrows) compared to wild-type (WT) mice. (**B and C**) Quantitative analysis of crypt depth in the ileum (**B**) and muscularis thickness in the colon (**C**) is provided (*n* = 4). (**D**) The relative expression levels of epithelial lineage marker genes (Lgr5, Alpi, Clca1, Muc2, Chga, Vil1) in the ileum are shown (*n* = 5). (**E**) There is a downregulation of bile acid transporter genes (Slc10a2) in Tet2-iKO mice (*n* = 5). (**F**) In Caco-2 cells, Tet2 knockdown or inhibition using the Tet2 inhibitor Bobcat 339 results in decreased ASBT protein levels, whereas Tet2 activation via ascorbic acid leads to increased ASBT expression. (**G**) Dot blot analysis of 5hmC levels in control versus Tet2-knockdown Caco-2 cells (*n* = 3). (**H**) Chromatin immunoprecipitation (ChIP)-qPCR showing Tet2 enrichment at the *Slc10a2* promoter region in Caco-2 cells; IgG was used as a control (*n* = 3). (**I**) Young Tet2-iKO mice exhibit increased fecal bile acid excretion and elevated bile acid levels in plasma and liver compared to WT littermates (*n* = 5). (**J**) Hepatic expression of the bile acid synthesis enzyme Cyp27a1 (*n* = 4). (**K**) Metabolomic profiling reveals elevated fecal hyocholic acid (HCA) in Tet2-iKO mice. (**L**) Molecular docking predicts favorable binding of HCA within the ASBT binding site. (**M**) Surface plasmon resonance (SPR) confirms direct binding between HCA and ASBT. (**N and O**) 16S rRNA sequencing shows altered microbiota composition and increased abundance of *Lactobacillus* in Tet2-iKO mice (*n* = 3). (**P**) *In vitro* bacterial growth assay demonstrates the dose-dependent effect of HCA on *Lactobacillus* proliferation. Differences between the two groups were assessed by Student’s *t*-test; multiple groups were compared by one-way ANOVA. Alpha-diversity indices (ACE, Chao1, Simpson) were analyzed using the Wilcoxon rank-sum test. Data are mean ± SEM, *P < 0.05, **P < 0.01, ***P < 0.001, ****P < 0.0001.

The successful deletion of Tet2 was confirmed through reductions in both mRNA (Fig. S1A through C) and protein expression (Fig. S1K). Although no discernible differences were observed in gross morphology, body weight, or intestinal length between Tet2-iKO and WT mice (Fig. S1D and E), histological examination uncovered significant architectural abnormalities in the knockout mice, including submucosal gaps at villus tips (red arrows, Fig. 1A) and regions of epithelial erosion with exposed lamina propria in the colon (black arrows, Fig. 1A). While mucosal layer height, villus length, and villus width were unchanged compared to controls (Fig. S1F through H), crypt depth and muscularis thickness were markedly reduced in Tet2-iKO mice (Fig. 1B and C). Proliferation activity, as assessed by Ki-67 staining, showed no significant change (Fig. S1I). Subsequently, we investigated markers of epithelial cell lineage (7). The expression of genes, such as Lgr5, Alpi, Clca1, Muc2, Chga, and Vil1, was significantly altered in the KO ileum (Fig. 1D), indicating impaired differentiation. Additionally, genes associated with barrier function, including Zo-1, Claudin-1, Occludin-1, and E-cadherin, exhibited downregulation at the mRNA level (Fig. S1J), and a reduction in ZO-1 protein levels was confirmed (Fig. S1K). These findings suggest that Tet2 deficiency compromises epithelial identity and barrier integrity, which may predispose individuals to intestinal disorders.

RNA-seq on Tet2-iKO intestinal crypts revealed a significantly suppressed bile acid transporter gene, *Slc10a2* (ASBT) (Fig. 1E; Fig. S2A through C). ASBT is a principal transporter governing bile acid pool size and metabolic homeostasis (8). Overall transcriptomic analysis showed 76 downregulated and 7 upregulated genes in the mutant mice (Fig. S2A and B). This regulatory relationship was further corroborated in Caco-2 cells, where both Tet2 knockdown and pharmacological inhibition with Bobcat-339 reduced ASBT expression, whereas Tet2 activation via ascorbic acid enhanced it (9), establishing Tet2 as a positive regulator of ASBT (Fig. 1F).

To assess the effect of Tet2 deficiency on global DNA hydroxymethylation, we measured 5hmC levels in Tet2-knockdown Caco-2 cells. 5hmC was significantly reduced, confirming impaired hydroxymethylation upon Tet2 loss (Fig. 1G). ChIP-qPCR showed Tet2 enrichment at the SLC10A2 promoter (–212 to –70 bp), indicating direct binding (Fig. 1H).

Consistent with ASBT downregulation, Tet2-iKO mice exhibited disrupted bile acid homeostasis, including increased fecal and systemic bile acids (Fig. 1I) and upregulation of Cyp27a1 without changes in Cyp7a1/b1/8b1 (Fig. 1J) (10). Metabolomics revealed HCA accumulation in KO feces (Fig. 1K; Fig. S2D). Primary bile acids increased while secondary species decreased in KOs (Fig. S2E and F), a profile mirrored by ASBT inhibitor treatment (Fig. S3) (11), supporting impaired bile acid reabsorption. Molecular docking suggested ASBT–HCA binding (Fig. 1L), confirmed by SPR with measurable affinity (Fig. 1M). Given bile acids as host–microbiota mediators (12), we assessed microbial changes. 16S sequencing showed distinct communities in KOs, with reduced α-diversity and altered β-diversity (Fig. S4A through D) (7), a shifted Firmicutes/Bacteroidetes ratio (Fig. S4E), and enriched bile salt hydrolase (BSH)-active *Lactobacillus* in young KOs (Fig. 1N and O) (13). *In vitro*, HCA had concentration-dependent effects on *L. salivarius* growth (Fig. 1P).

**Fig 2:**
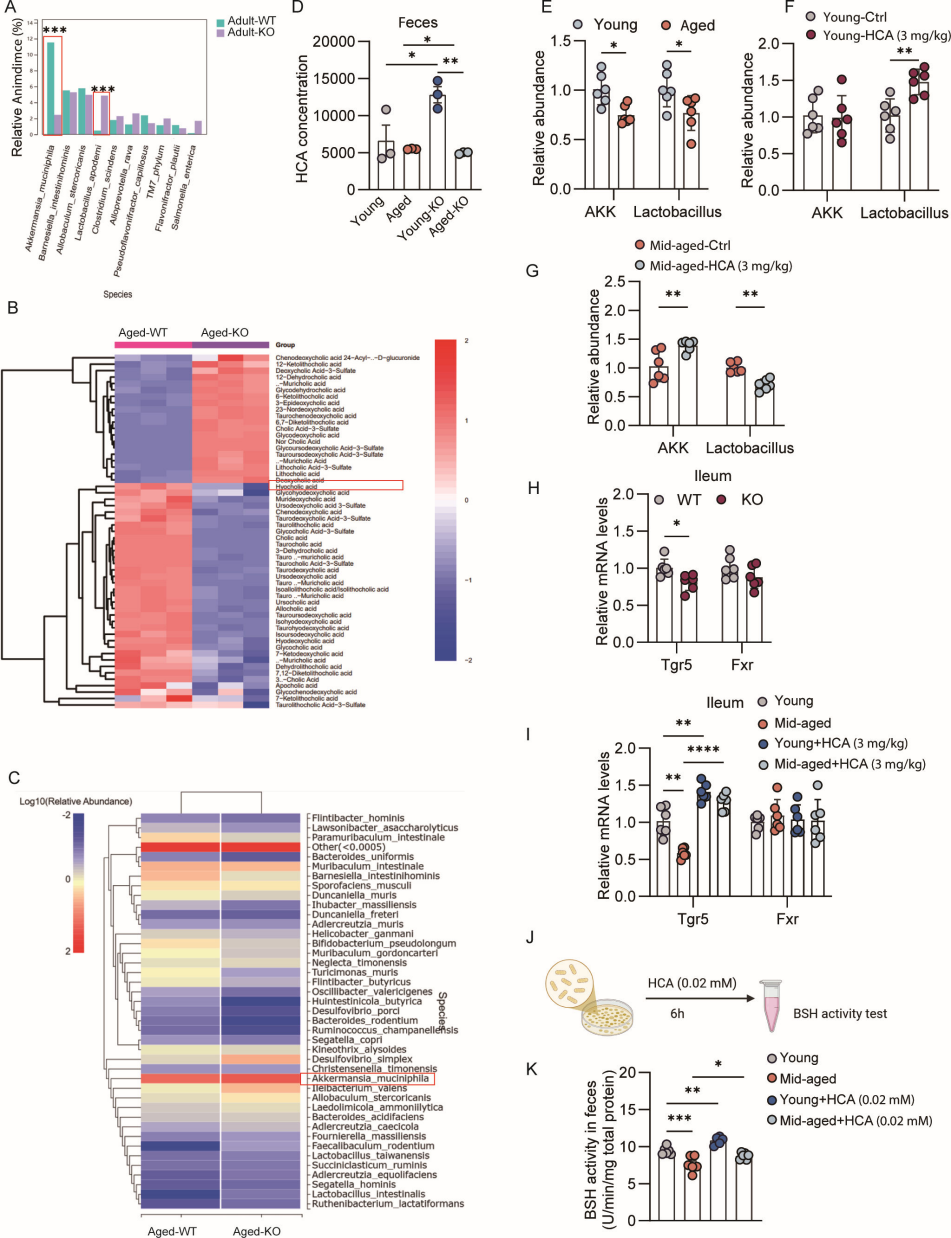
Tet2 deletion and HCA administration alter gut microbiota composition in an age-dependent manner. (**A**) Fecal microbiota analysis in middle-aged (5-month-old) Tet2-iKO and wild-type (WT) mice, showing sustained enrichment of *Lactobacillus* and reduction of *Akkermansia muciniphila* (AKK) in knockout mice. (**B**) Fecal HCA levels are reduced in aged Tet2-iKO mice compared to age-matched WT controls (*n* = 3). (**C**) In contrast to younger mice, aged (12-month-old) Tet2-iKO mice exhibit decreased *Lactobacillus* and increased AKK. (**D**) Tet2 deletion significantly reduces fecal HCA levels in both young and aged mice (*n* = 3). (**E**) Relative abundance of AKK and *Lactobacillus* in young versus aged mice (*n* = 6). (**F**) HCA treatment in young mice increases *Lactobacillus* and decreases AKK (*n* = 6). (**G**) In middle-aged mice, HCA administration enriches AKK while suppressing *Lactobacillus*, indicating an age-dependent microbial response (*n* = 6). (**H**) Intestinal expression of Fxr and Tgr5 is downregulated in young Tet2-iKO mice (*n* = 6). (**I**) Effects of aging and HCA treatment on bile acid receptor expression (*n* = 6). (**J**) Schematic of the *ex vivo* fecal incubation assay. Fecal samples from young and aged mice were cultured and treated with HCA, followed by BSH activity measurement. (**K**) BSH activity in HCA-treated fecal cultures (*n* = 6). For multi-group comparisons, two-way ANOVA with Tukey’s or Sidak’s post hoc test was used. Data are mean ± SEM, *P < 0.05, **P < 0.01, ***P < 0.001, ****P < 0.0001.

We previously reported age-related declines in *Lactobacillus* and *Akkermansia muciniphila* (AKK) (14). Tet2 deficiency altered this trend: 5-month-old KOs had elevated *Lactobacillus* but reduced AKK (Fig. 2A), while 12-month-old KOs showed decreased HCA, reduced *Lactobacillus*, and increased AKK (Fig. 2B and C). Tet2 deletion consistently lowered fecal HCA in both ages (Fig. 2D).

HCA administration caused age-dependent shifts: it promoted *Lactobacillus* and suppressed AKK in young mice, but enriched AKK while inhibiting *Lactobacillus* in middle-aged mice (Fig. 2F and G). BSH-active genera (e.g., *Lactobacillus*, *Bifidobacterium*) are enriched in young KOs. This HCA effect reversal suggests aging remodels host–microbe crosstalk, potentially via age-altered bile acid receptors (15, 16). Tet2 deletion downregulated both Fxr and Tgr5 in young mice (Fig. 2H). Tgr5 expression varied with age and was upregulated by HCA treatment, whereas Fxr remained largely unchanged (Fig. 2I). In contrast, HCA had minimal effect on Fxr transcription, consistent with its primary regulation at the ligand and post-transcriptional levels. Fecal BSH activity was reduced in middle-aged mice (Fig. 2J and K). While low-dose HCA enhanced BSH activity in young mice—likely by enriching BSH-positive Lactobacillus—it had a limited effect in aged mice with fewer *Lactobacillus* (Fig. 2E and K), indicating that HCA’s impact depends on the baseline abundance of BSH-capable bacteria. Aging also reshapes the gut microenvironment in ways that could explain these dynamics (17, 18). In addition, age-related mucosal thinning (19, 20) and immune decline (21, 22) may further alter ecological niche availability.

In conclusion, our study establishes the DNA demethylase Tet2 as a key regulator of intestinal epithelial homeostasis. Tet2 deficiency disrupts tissue architecture, impairs barrier function, and suppresses bile acid transport, leading to the accumulation of specific bile acids, such as HCA. This metabolic shift drives a reorganization of the gut microbiota, favoring bacteria with BSH activity. Our work thus reveals an epigenetic pathway linking Tet2 to bile acid metabolism and host–microbiota crosstalk, suggesting that dysregulation of this “bile acid–microbiota” axis may underlie age-related intestinal decline. Several limitations remain, including the need to fully elucidate how Tet2 controls bile acid–related genes and HCA levels, to functionally validate the proposed metabolic and microbial interactions, and to further explore the influence of aging on Tet2-mediated functions.

### Mice and genotyping

Intestinal epithelial-specific Tet2 knockout mice (Tet2-iKO) were generated by crossing Tet2fl/fl mice with Villin-Cre mice (obtained from Cyagen Biosciences). All experiments utilized male C57BL/6 mice at 6–8 weeks of age. All mice were maintained under specific pathogen-free conditions in a controlled environment (22°C ± 1°C, 55% ± 5% humidity, 12-h light/dark cycle) with *ad libitum* access to autoclaved water and standard chow diet. Genotyping was performed using PCR amplification of tail DNA with the following primers: Tet2-flox forward: 5′-GTG TTG AAT CAT TTC ACC TGC ATC-3′, Tet2-flox reverse: 5′-GAA AAC CCA GAA ACC CCT GAA CTA-3′; Villin-Cre forward: 5′-GTG TTT GGT TTG GTT TCC TCT GCA TAA GA-3′, Villin-Cre reverse: 5′-GCA GGC AAA TTT TGG TGT ACG GTC A-3′. All animal procedures were approved by the Tongji University Institutional Animal Care and Use Committee and adhered to ethical guidelines for preclinical research.

### Histological analysis and immunofluorescence

Intestinal tissues (proximal jejunum, distal ileum, and proximal colon) were collected and fixed in 4% paraformaldehyde for 24 h, embedded in paraffin, and sectioned at 5 μm thickness. Sections were stained with H&E using standard protocols. For immunofluorescence, sections were subjected to antigen retrieval in citrate buffer (pH 6.0) and incubated with primary antibodies against ZO-1 (1:200, Invitrogen, 33-9100), Ki-67 (1:100, Cell Signaling Technology, 9129S), and Tet2 (1:150, Cell Signaling Technology, 18,950S) overnight at 4°C, followed by incubation with appropriate fluorescent secondary antibodies (1:500, Invitrogen). Images were acquired using a Nikon Eclipse Ti2 microscope and analyzed with NIS-Elements software. Morphometric measurements (villus height, crypt depth, mucosal thickness) were performed on at least 10 well-oriented crypt-villus units per sample using ImageJ software (NIH).

### Cell culture and treatments

Human intestinal epithelial Caco-2 cells (ATCC HTB-37) were maintained in DMEM (Gibco) supplemented with 10% fetal bovine serum (FBS, Gibco), 1% non-essential amino acids, and 1% penicillin-streptomycin at 37°C in 5% CO_2_. For pharmacological modulation, cells were treated with Tet2 inhibitor Bobcat 339 (10 μM, MedChemExpress, HY-111558) or Tet2 activator ascorbic acid (100 μM, Sigma-Aldrich, A4544) for 24 h. For bile acid treatment experiments, cells were treated with hyocholic acid (HCA, 0–200 μM, MedChemExpress, HY-121238) for 24 h.

### Isolation of intestinal epithelial cells

The small intestine was opened longitudinally and thoroughly washed with ice-cold PBS supplemented with penicillin (200 μg/mL) and streptomycin (200 μg/mL). The tissue was then cut into approximately 5 mm segments. These segments were incubated in PBS containing 2 mM EDTA on ice for 1 h. Subsequently, the tissues were transferred to fresh ice-cold PBS and vigorously shaken for 2 min to dissociate epithelial cells (including both villi and crypts). The resulting cell suspension was centrifuged, and the pellet containing intestinal epithelial cells was collected for further analysis.

### RNA extraction and quantitative real-time PCR

Total RNA was extracted from intestinal tissues or cells using TRIzol reagent (Invitrogen) according to the manufacturer’s instructions. RNA quality was assessed using NanoDrop 2000 (Thermo Scientific) and agarose gel electrophoresis. cDNA was synthesized from 1 μg total RNA using HiScript III All-in-one RT SuperMix Perfect (Vazyme Biotech Co., Ltd.). Quantitative real-time PCR was performed using ChamQ Universal SYBR qPCR Master Mix (Vazyme Biotech Co., Ltd.) on a QuantStudio 7 Flex Real-Time PCR System (Thermo Fisher Scientific). The PCR conditions were 95°C for 30 s, followed by 40 cycles of 95°C for 5 s and 60°C for 30 s. Gene expression was normalized to β-actin expression and calculated using the 2^−ΔΔCt^ method. Primer sequences are listed in Table S1.

### Chromatin immunoprecipitation-qPCR

ChIP was performed using the BeyoChIP Enzymatic ChIP Assay Kit (Beyotime, Cat. No. P2083S) following the manufacturer’s protocol. In brief, cells were crosslinked with 1% formaldehyde for 10 min at room temperature, and the reaction was quenched with 125 mM glycine. After nuclei extraction, chromatin was enzymatically fragmented to an average size of 200–1,000 bp. Lysates were immunoprecipitated with 2–5 μg of Tet2 antibody (Santa Cruz, GTX124205) or control IgG, followed by capture with Protein A/G magnetic beads. After washing, bound complexes were eluted, reverse cross-linked, and the DNA was purified. Enriched DNA was quantified by qPCR using the following primers targeting the SLC10A2 promoter: forward: 5′-CGTGGCTATTTCTGTGTGGC-3′; reverse: 5′-TGTCAGTTTCGAGAGGCTGC-3′. All ChIP-qPCR results were normalized to input DNA and expressed as a percentage of input.

### Western blotting

Cells were lysed in ice-cold lysis buffer supplemented with protease inhibitors and sonicated on ice. The lysates were centrifuged at 12,000 × *g* for 15 min at 4°C, and the protein concentration was determined using the Bradford assay (Bio-Rad, Hercules, CA, USA). Equal amounts of protein (20 μg) were separated by 10%–12% SDS-PAGE and electrophoretically transferred to PVDF membranes. The membranes were blocked with 5% skim milk in TBST (tris-buffered saline containing 0.1% Tween-20) for 1 h at room temperature, followed by incubation with primary antibodies at 4°C overnight. After washing, the membranes were incubated with corresponding HRP-conjugated secondary antibodies at room temperature for 1 h. Protein bands were detected using a Tanon 5200CE chemiluminescence imaging system (Tanon, Shanghai, China).

### RNA sequencing and bioinformatics analysis

Total RNA was extracted from intestinal epithelial cells isolated via the EDTA-chelation method as previously described. Sequencing libraries were constructed using the BGI Proprietary RNA Library Preparation Kit and subjected to paired-end sequencing (50 bp) on the BGISEQ-500 platform. Raw reads were assessed for quality using FastQC, and then aligned to the mouse reference genome (GRCm39) with the STAR aligner. Gene expression levels were quantified using featureCounts, and differential expression analysis was performed with DESeq2. Significantly differentially expressed genes were identified based on the thresholds: adjusted *P* value <0.05 and |log₂(fold change)| >1. Gene Ontology enrichment analysis was carried out using the clusterProfiler R package.

### Bile acid quantification by UHPLC–MS/MS

Total bile acid (TBA) levels in fecal, plasma, and liver samples were measured using a commercial TBA Assay Kit (Nanjing Jiancheng Bioengineering Institute, #E003-1-1) per the manufacturer’s protocol. For targeted bile acid profiling, samples were prepared via extraction with 200 μL of acetonitrile:methanol (1:1, vol:vol) containing a mixture of isotopically-labeled internal standards. Chromatographic separation was performed on a Waters ACQUITY UPLC BEH C18 column (150 × 2.1 mm, 1.7 μm) maintained at 45°C using a Vanquish UHPLC system (Thermo Fisher Scientific). The mobile phase consisted of (i) 5 mmol/L ammonium acetate in water and (ii) acetonitrile. An injection volume of 1 μL was used, with the auto-sampler temperature set at 4°C.

Mass spectrometric detection was carried out on an Orbitrap Exploris 120 instrument (Thermo Fisher Scientific) operated in parallel reaction monitoring mode. Due to the lack of detectable product ions for most analytes, quantification was based on high-resolution precursor ions. Ion source parameters were as follows: spray voltage, ±3,500/−3,200 V; sheath gas, 40; auxiliary gas, 15; capillary temperature, 320°C. Calibration curves and limits of quantification are provided in Table S2.

### 16S rRNA gene sequencing and microbiota analysis

Fecal DNA was isolated with the QIAamp PowerFecal Pro DNA Kit (Qiagen) following the supplier’s protocols. The V3–V4 region of the 16S rRNA gene was amplified and processed for sequencing in accordance with the Illumina 16S Metagenomic Sequencing Library Guide (Illumina, San Diego, CA, USA). Sequencing was performed on an Illumina MiSeq platform with a 2 × 250-bp reagent kit at the Teagasc Sequencing Facility. Paired-end reads were merged using FLASH. Subsequent processing, including quality filtering (Q-score > 25), removal of barcode errors, and elimination of short sequences, was conducted in QIIME2 with DADA2 denoising. Denoising, chimera filtering, and operational taxonomic unit (OTU) clustering were carried out with USEARCH (v7.0.1090). OTUs were aligned with PyNAST and taxonomically classified using BLAST against the SILVA SSURef database (release 123). Bacterial diversity—both alpha and beta—was assessed within QIIME. Oscillatory patterns of OTUs were evaluated using the JTK_Cycle algorithm. The average sequencing depth was approximately 81,000–85,000 raw reads per sample, yielding 77,000–78,000 clean reads after quality control (*n* = 3 biologically independent replicates per group).

### Molecular docking and surface plasmon resonance

Molecular docking analysis was carried out with AutoDock Vina (v1.2.0). SPR assays were performed on an OpenSPR instrument (Nicoya Lifesciences). Recombinant human ASBT protein (Proteintech, #Ag18808) was immobilized onto a COOH sensor chip. HCA solutions (0.78–100 μM) were infused at a flow rate of 20 μL/min with a contact time of 240 s, followed by a dissociation period of 360 s. Sensorgram data were processed and fitted using TraceDrawer software (Ridgeview Instruments ab, Sweden).

### Bacterial strains and culture conditions

Lactobacillus_salivarius was obtained from the American Type Culture Collection (ATCC). The bacterium was cultured in de Man, Rogosa, and Sharpe broth, prepared by dissolving 66.2 g of the powder in 1 L of distilled water, followed by aliquoting and sterilization via autoclaving at 121°C for 15 min. To evaluate the effect of HCA on bacterial growth, cultures were inoculated into media supplemented with 0.02 or 0.2 mM HCA. Bacterial growth was monitored by measuring the optical density at 600 nm (OD₆₀₀) every 4 h using a microplate reader. The initial OD₆₀₀ of each culture was adjusted to 0.1. The growth rate was calculated as (OD_n_ − OD_0_)/OD_0_, where OD_0_ represents the initial absorbance, and OD_n_ denotes the absorbance after n hours of HCA treatment.

### BSH activity in the feces

Fecal BSH activity was measured using a commercial assay kit (Shanghai Yanjin Biotechnology) according to the manufacturer’s instructions. Briefly, frozen fecal samples (250 mg) were homogenized in liquid nitrogen and suspended in 5 mL of ice-cold PBS. After incubation on ice for 10 min, bacterial cells were collected from the supernatant by centrifugation at 7,000 × *g* for 15 min at 4°C. The pellet was washed with PBS and lysed via sonication (60 s). The lysate was centrifuged at 9,700 × *g* for 15 min, and the resulting supernatant was used for the enzymatic assay. BSH activity was determined by measuring liberated taurine at 570 nm, with a unit (U/mL) defined as the amount of enzyme releasing 1 μmol taurine per minute. Total protein concentration was determined using a BCA assay (Thermo Fisher Scientific) for normalization.

### Statistical analysis

Statistical analysis was performed using GraphPad Prism 9.0. Normality was assessed using the Shapiro-Wilk test. For comparisons between two groups, the unpaired Student’s *t*-test or Mann-Whitney U test was used as appropriate. For multiple comparisons, one-way or two-way ANOVA, followed by Tukey’s or Sidak’s post hoc test, was used. All data show mean ± SEM. *P* < 0.05 was considered statistically significant.

## Data Availability

All data are included in the main text or the Supplementary material. The RNA-seq data generated in this study have been deposited in the NCBI Gene Expression Omnibus (GEO) under accession number GSE307332. The original 16S rRNA sequencing data are available in the NCBI Sequence Read Archive (SRA) under accession number PRJNA1315213.

## SUPPLEMENTAL MATERIAL

Supplemental material mbio.03562-25-s0001.docx Fig. S1 to S4; Tables S1 and S2.

ASM does not own the copyrights to Supplemental Material that may be linked to, or accessed through, an article. The authors have granted ASM a non-exclusive, world-wide license to publish the Supplemental Material files. Please contact the corresponding author directly for reuse.
