# Supplementary material for: Intestinal epithelial Tet2 deficiency reprograms the gut microbiota through bile acid metabolic alterations
Source: mBio. 2026 Jan 26;17(3):e03562-25. doi: 10.1128/mbio.03562-25 (PMC12977613; doi:10.1128/mbio.03562-25)
Supplement: Supplemental material — Fig. S1 to S4; Tables S1 and S2. [file mbio.03562-25-s0001.docx]

**Supplemental Information**

**
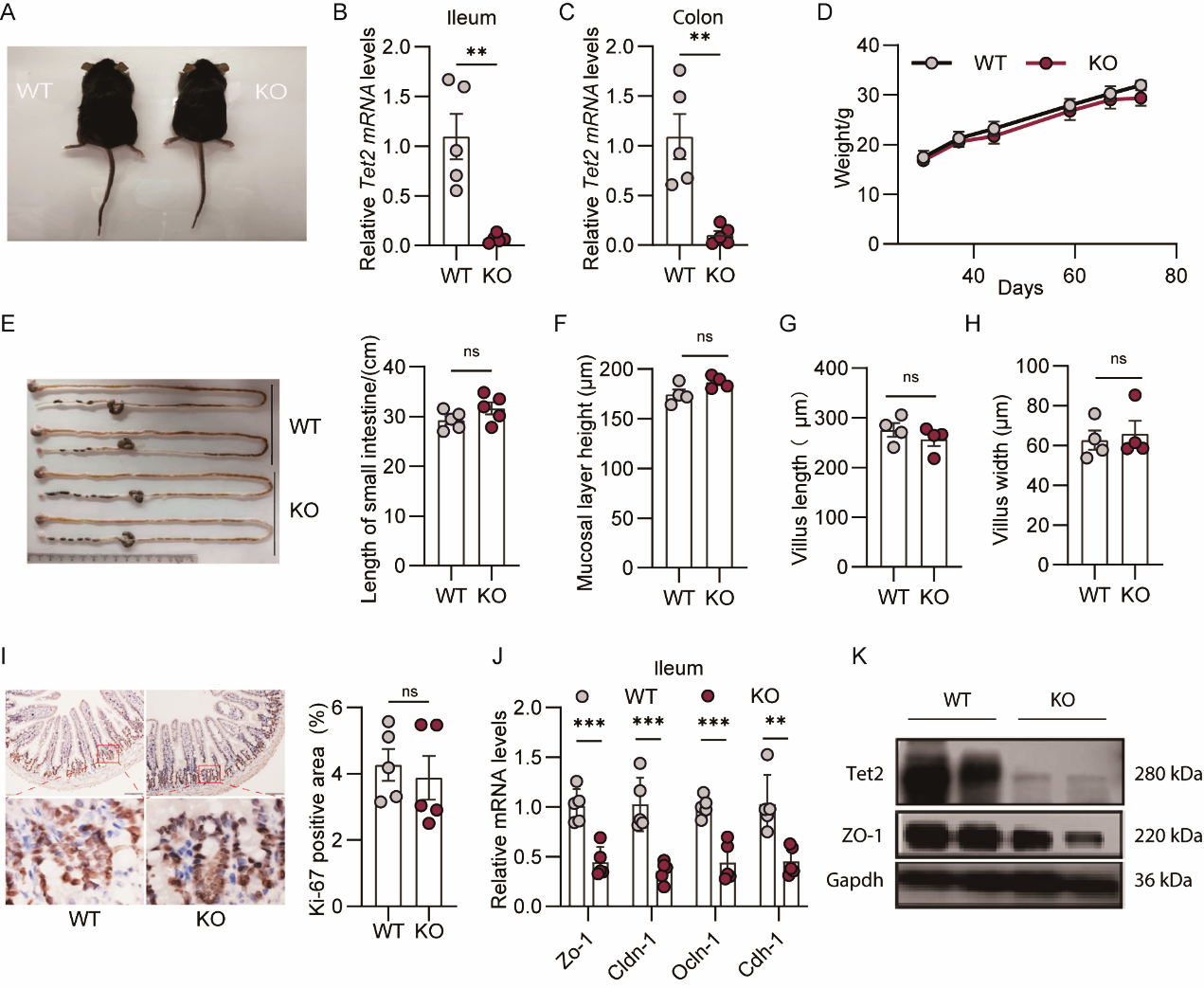
**

**Figure S1. Validation of intestinal Tet2 deletion and baseline phenotyping.** (A) Gross morphology of WT and TET2-iKO mice. (B–C) Verification of Tet2 deletion at mRNA level in intestinal tissues (*n* = 5). (D–E) Body weight (D) and intestinal length (E) of WT and TET2-iKO mice (*n* = 5). (F–H) Quantification of ileal villus length (F), villus width (G), and colonic mucosal thickness (H) (*n* = 4). (I) Ki-67 staining showing comparable epithelial proliferation between groups (*n* = 5). (J–K) Reduced expression of tight junction genes and decreased protein levels of ZO-1 in TET2-iKO ileum (*n* = 5). Data are mean ± SEM, **p<0.01, ***p<0.001; statistics as indicated in Methods. The overall significance between the two groups was determined by Student’s t-test. The overall significance among three or four groups was determined by one-way ANOVA.

**
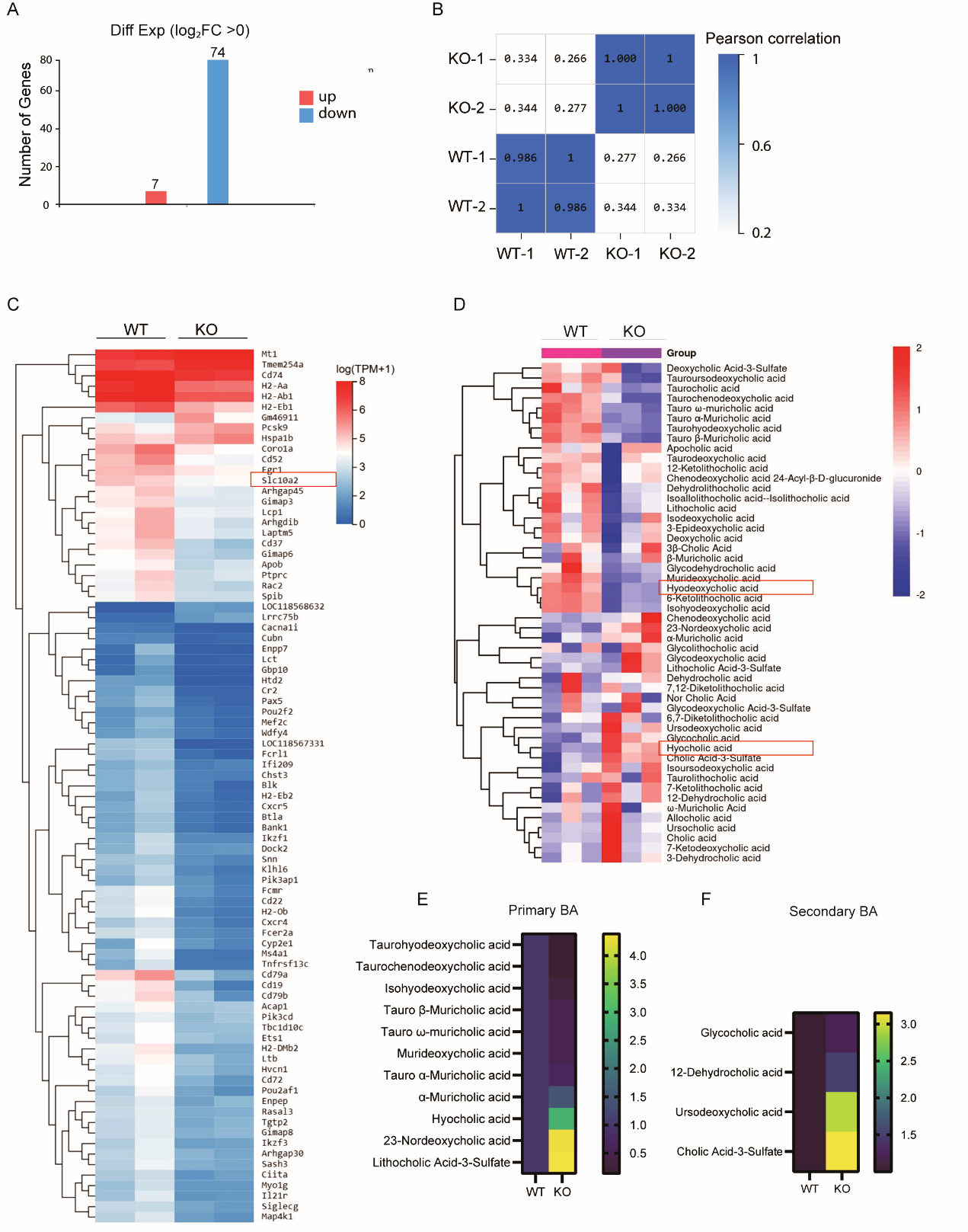
**

**Figure S2. Transcriptomic and metabolomic profiling of Tet2-deficient intestinal epithelium.** (A) Volcano plot of differentially expressed genes between WT and Tet2-iKO mice. (B) Sample correlation analysis shows high within-group consistency and clear separation by genotype. (C) Expression of the bile acid transporter Slc10a2/ASBT is downregulated in Tet2-iKO mice (*n* = 2). (D) Untargeted fecal metabolomics reveals alterations in bile acid composition (*n* = 3). (E, F) Heatmaps display relative levels of primary (E) and secondary (F) bile acids in fecal samples from control and Tet2-iKO mice. Tet2 deficiency increases most primary bile acids but decreases the majority of secondary bile acids.

**
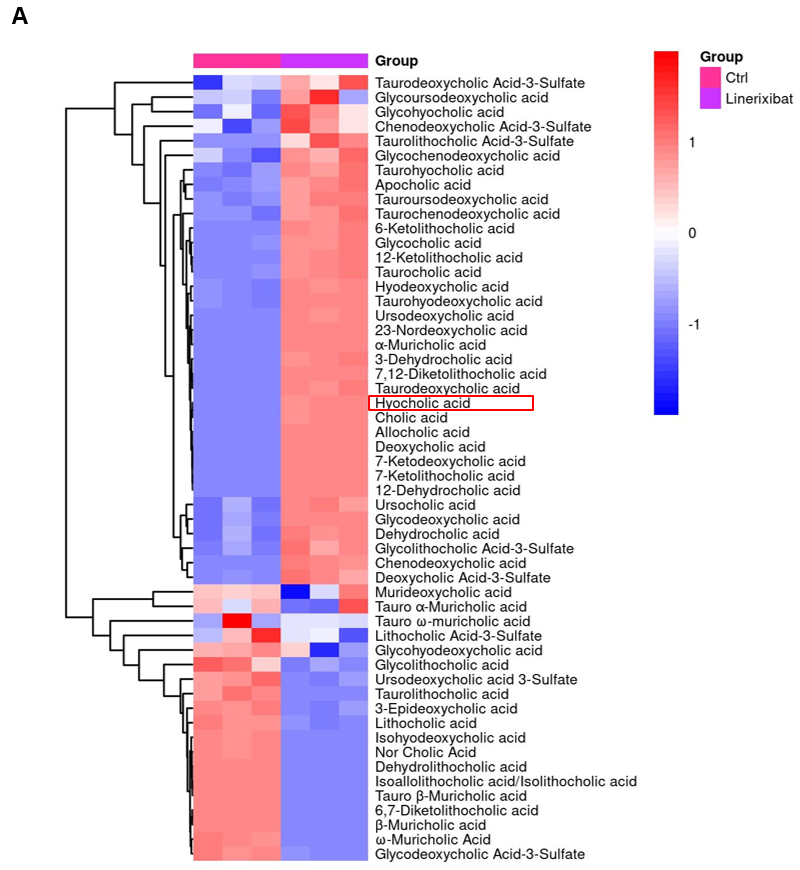
**

**Figure S3. Pharmacological inhibition of ASBT alters fecal bile acid composition.** (A) Treatment with the ASBT inhibitor Linerixibat increases fecal HCA abundance (*n* = 3).

**
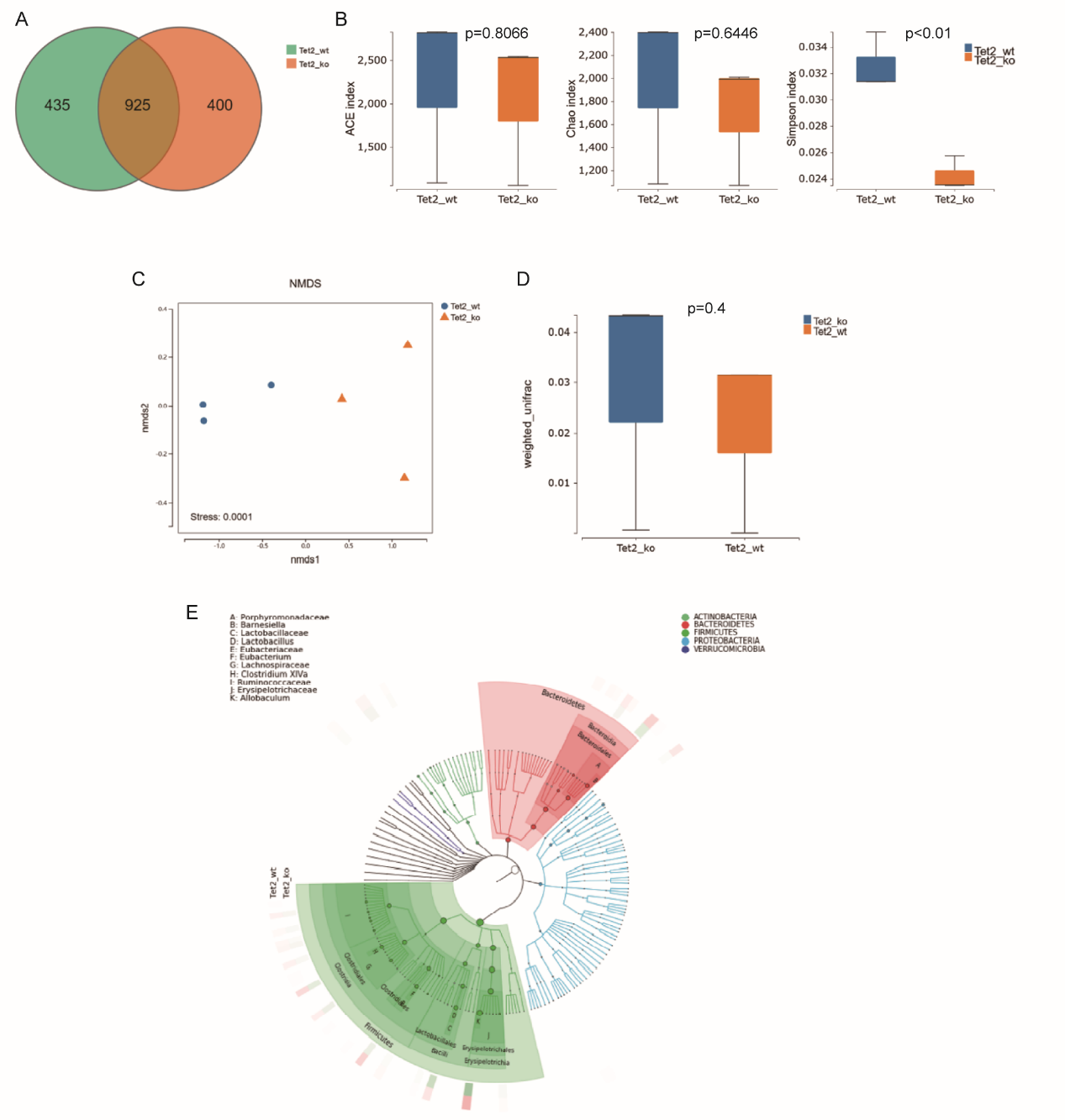
**

**Figure S4. Gut microbiota alterations in Tet2-iKO mice.** (A) Taxonomic composition at the phylum level. (B) α-Diversity indices (ACE, Shannon, Chao1) indicate reduced microbial richness and evenness in Tet2-iKO mice. (C) Altered community stability in Tet2-iKO mice. (D) β-Diversity analysis shows distinct clustering by genotype. (E) GraPhlAn phylogenetic tree illustrating taxonomic structure and relative abundances, with an altered Firmicutes/Bacteroidetes ratio in knockout mice.

**Table S1. Primer sequences used in this study.**

| **Target gene** | | **Forward primer (5'--3')** | **Reverse primer (5'--3')** |
| --- | --- | --- | --- |
| **qPCR** | Tet2 | TTGTTCCTGTTCCGCTACG | TGAGGTCTCTCTGCTAACGG |
|  | Lgr5 | CAGGTCAATACCGGAGCGAG | GCGAGGCACCATTCAAAGTC |
|  | Alpi | AGGACATCGCCACTCAACTC | GGTTCCAGACTGGTTACTGTCA |
|  | Clca1 | CGACGTGCCGGAAGATGAA | GCTCTCGGGAATCAAAATGGC |
|  | Muc2 | GAAGCCAGATCCCGAAACCA | GAATCGGTAGACATCGCCGT |
|  | Chga | AGACTACAGACCCACTCCCG | AGATGACTTCCAGGACGCAC |
|  | Vil1 | GCTTGCCACAACTTCCTAAGAT | TCAGTTTAGTCATGGTGGACGA |
|  | Zo1 | TGGTGGAAATGATGTCGGAATA | CTTTAGGGAGGTCAAGGAGGAA |
|  | Slc10a2 | GCGACATGGACCTCAGTGTT | TTCCCGAGTCAACCCACATC |
|  | Cyp7a1 | AAACCCTCCAGGGAGATGCT | CCGGCTTCAAACATCACTCG |
|  | Cyp27a1 | TGCCTGGGTCGGAGGAT | GAGCCAGGGCAATCTCATACTT |
|  | Cyp8b1 | CTGGCTTCCTGAGCTTATTC | CCCAGTAGGGAGTAGACAAA |
|  | Cyp7b1 | GGAGCCACGACCCTAGATG | GCCATGCCAAGATAAGGAAGC |
|  | Tgr5 | CACTGCTCTTCTTGCTGTGTTGG | GAGCGATAACAGAGTTCCAGGC |
|  | Fxr | GGGATGAGTGTGAAGCCAGCTA | GTGGCTGAACTTGAGGAAACGG |
|  | β-actin | GAGGTATCCTGACCCTGAAGTA | CACACGCAGCTCATTGTAGA |

**Table S2. Summary of calibration curves, lower limits of detection (LLODs), and lower limits of quantification (LLOQs) for the quantified bile acids.**

internal standards：

| **Number** | **Name** | **Abbreviation** | **Molecular formula** |
| --- | --- | --- | --- |
| BIS-1 | [2H4]-Lithocholic Acid | LCA-D4 | C24D4H3603 |
| BIS-2 | [2H6]-Deoxycholic Acid | DCA-D6 | C24D6H3404 |
| BIS-3 | [2H4]-Lithocholic Acid-3-Sulfate | LCA-3S-D4 | C24D4H34O6S.2Na |
| BIS-4 | [2H4]-Glycocholic acid | GCA-D4 | C26D4H39NO6 |
| BIS-5 | [2H4]-Taurochenodeoxycholic Acid | TCDCA-D4 | C26D4H40NO6S·Na |
| BIS-6 | [2H4]-Glycocholic Acid-3-Sulfate | GCA-3S-D4 | C26H37D4NNa2O9S |

calibration curves：

| **Name of Substance** | **RT（min）** | **Equation of a line** |
| --- | --- | --- |
| 01_Dehydrolithocholic acid | 22 | Y = 0.0117X + 0.0263; R^2: 0.998, weight: 1/X^1 |
| 02_Isoallolithocholic acid/Isolithocholic acid | 21.6 | Y = 0.0345X + 0.0344; R^2: 0.997, weight: 1/X^1 |
| 03_Lithocholic acid | 21.92 | Y = 0.0099X + 0.0437; R^2: 0.997, weight: 1/X^1 |
| 04_23-Nordeoxycholic acid | 19.76 | Y = -1.40e-05X^2 + 0.0116X + 0.0011; R^2: 1.000, weight: 1/X^1 |
| 05_6-Ketolithocholic acid | 19.26 | Y = 0.0105X + 0.0106; R^2: 0.997, weight: 1/X^1 |
| 06_7-Ketolithocholic acid | 19.83 | Y = 0.0054X + 0.0041; R^2: 0.998, weight: 1/X^1 |
| 07_12-Ketolithocholic acid | 19.95 | Y = 0.0059X + 0.0016; R^2: 0.998, weight: 1/X^1 |
| 08_Apocholic acid | 20.09 | Y = 0.0030X + 0.0260; R^2: 0.998, weight: 1/X^1 |
| 09_Isoursodeoxycholic acid | 16.87 | Y = -4.88e-06X^2 + 0.0071X - 0.0025; R^2: 1.000, weight: 1/X^1 |
| 10_Murideoxycholic acid | 17.08 | Y = -5.98e-07X^2 + 0.0047X + 0.0131; R^2: 1.000, weight: 1/X^1 |
| 11_Isohyodeoxycholic acid | 18.39 | Y = 0.0070X + 0.0038; R^2: 0.998, weight: 1/X^1 |
| 12_Ursodeoxycholic acid | 19.03 | Y = 0.0073X + 0.0052; R^2: 0.999, weight: 1/X^1 |
| 13_Hyodeoxycholic acid | 19.27 | Y = 0.0097X + 0.0074; R^2: 0.999, weight: 1/X^1 |
| 14_3-Epideoxycholic acid | 19.52 | Y = -1.50e-06X^2 + 0.0096X + 0.0301; R^2: 0.999, weight: 1/X^1 |
| 15_Chenodeoxycholic acid | 20.81 | Y = 0.0108X + 0.1444; R^2: 0.998, weight: 1/X^1 |
| 16_Deoxycholic acid | 20.91 | Y = 0.0073X + 0.1082; R^2: 0.998, weight: 1/X^1 |
| 17_Isodeoxycholic acid | 21.52 | Y = 0.0492X + 0.0370; R^2: 0.998, weight: 1/X^1 |
| 18_Nor Cholic Acid | 13.19 | Y = -8.26e-07X^2 + 0.0064X + 0.0136; R^2: 0.999, weight: 1/X^1 |
| 19_Dehydrocholic acid | 5.91 | Y = 0.0065X + 0.0034; R^2: 0.997, weight: 1/X^1 |
| 20_7,12-Diketolithocholic acid | 6.69 | Y = -1.54e-06X^2 + 0.0044X + 0.0031; R^2: 1.000, weight: 1/X^1 |
| 21_6,7-Diketolithocholic acid | 19.72 | Y = 0.0033X + 0.0039; R^2: 0.998, weight: 1/X^1 |
| 22_7-Ketodeoxycholic acid | 15.02 | Y = 0.0087X + 0.0241; R^2: 0.998, weight: 1/X^1 |
| 23_12-Dehydrocholic acid | 15.34 | Y = 0.0056X - 0.0014; R^2: 0.998, weight: 1/X^1 |
| 24_3-Dehydrocholic acid | 18.37 | Y = 0.0038X + 0.0023; R^2: 0.998, weight: 1/X^1 |
| 25_Ursocholic acid | 8.32 | Y = 0.0043X + 0.0035; R^2: 0.997, weight: 1/X^1 |
| 26_3β-Cholic Acid | 12.12 | Y = 0.0178X + 0.0140; R^2: 0.999, weight: 1/X^1 |
| 27_ω-Muricholic Acid | 12.4 | Y = 0.0051X + 0.0669; R^2: 0.998, weight: 1/X^1 |
| 28_α-Muricholic acid | 13.38 | Y = 0.0046X + 0.0038; R^2: 0.999, weight: 1/X^1 |
| 29_β-Muricholic acid | 14.08 | Y = 0.0059X + 0.0036; R^2: 0.999, weight: 1/X^1 |
| 30_Hyocholic acid | 18 | Y = -5.43e-07X^2 + 0.0048X + 0.0082; R^2: 1.000, weight: 1/X^1 |
| 31_Allocholic acid | 18.95 | Y = 0.0085X + 0.0775; R^2: 0.998, weight: 1/X^1 |
| 32_Cholic acid | 19.09 | Y = 0.0069X + 0.0549; R^2: 0.996, weight: 1/X^1 |
| 33_Glycolithocholic acid | 20.86 | Y = 0.0185X + 0.0141; R^2: 0.997, weight: 1/X^1 |
| 34_Glycoursodeoxycholic acid | 17.9 | Y = -1.96e-06X^2 + 0.0049X + 0.0019; R^2: 1.000, weight: 1/X^1 |
| 35_Glycohyodeoxycholic acid | 18.16 | Y = -1.96e-07X^2 + 0.0030X + 0.0248; R^2: 0.999, weight: 1/X^1 |
| 36_Glycochenodeoxycholic acid | 19.79 | Y = 0.0073X + 0.0054; R^2: 0.998, weight: 1/X^1 |
| 37_Glycodeoxycholic acid | 20.05 | Y = 0.0052X + 0.0036; R^2: 0.996, weight: 1/X^1 |
| 38_Lithocholic Acid-3-Sulfate | 19.92 | Y = -4.31e-06X^2 + 0.0109X + 0.0101; R^2: 1.000, weight: 1/X^1 |
| 39_Glycodehydrocholic acid | 4.9 | Y = 0.0087X + 0.0065; R^2: 0.997, weight: 1/X^1 |
| 40_Glycohyocholic acid | 16.14 | Y = -1.57e-06X^2 + 0.0041X + 0.0020; R^2: 0.999, weight: 1/X^1 |
| 41_Glycocholic acid | 18.59 | Y = 0.0117X + 0.0089; R^2: 0.998, weight: 1/X^1 |
| 42_Ursodeoxycholic acid 3-Sulfate | 8.44 | Y = -1.49e-06X^2 + 0.0040X + 0.0022; R^2: 0.999, weight: 1/X^1 |
| 43_Chenodeoxycholic Acid-3-Sulfate | 18.27 | Y = 0.0064X + 0.0036; R^2: 0.996, weight: 1/X^1 |
| 44_Deoxycholic Acid-3-Sulfate | 18.43 | Y = 0.0065X + 0.0044; R^2: 0.997, weight: 1/X^1 |
| 45_Taurolithocholic acid | 20.96 | Y = 0.0251X + 0.0333; R^2: 0.998, weight: 1/X^1 |
| 46_Cholic Acid-3-Sulfate | 10.69 | Y = 0.0078X + 0.0076; R^2: 0.997, weight: 1/X^1 |
| 47_Tauroursodeoxycholic acid | 18.56 | Y = 0.0083X + 0.0041; R^2: 0.996, weight: 1/X^1 |
| 48_Taurohyodeoxycholic acid | 18.7 | Y = -4.08e-06X^2 + 0.0129X + 0.0074; R^2: 0.999, weight: 1/X^1 |
| 49_Taurochenodeoxycholic acid | 20.05 | Y = -4.79e-07X^2 + 0.0044X + 0.0204; R^2: 1.000, weight: 1/X^1 |
| 50_Taurodeoxycholic acid | 20.29 | Y = -6.24e-06X^2 + 0.0165X + 0.0120; R^2: 0.999, weight: 1/X^1 |
| 51_Glycolithocholic Acid-3-Sulfate | 18.62 | Y = 0.0054X + 0.0017; R^2: 0.997, weight: 1/X^1 |
| 52_Tauro ω-muricholic acid | 12.15 | Y = 0.0113X + 0.0089; R^2: 0.998, weight: 1/X^1 |
| 53_Tauro α-Muricholic acid | 13.15 | Y = 0.0033X + 0.0026; R^2: 0.998, weight: 1/X^1 |
| 54_Tauro β-Muricholic acid | 13.46 | Y = 0.0074X + 0.0188; R^2: 0.999, weight: 1/X^1 |
| 55_Taurohyocholic acid | 17.63 | Y = 0.0139X - 0.0005; R^2: 0.997, weight: 1/X^1 |
| 56_Taurocholic acid | 18.91 | Y = -4.66e-07X^2 + 0.0040X + 0.0089; R^2: 1.000, weight: 1/X^1 |
| 57_Glycoursodeoxycholic Acid-3-Sulfate | 4.28 | Y = 0.0046X + 0.0034; R^2: 0.997, weight: 1/X^1 |
| 58_Glycochenodeoxycholic Acid 3-Sulfate | 15.31 | Y = -3.36e-07X^2 + 0.0035X + 0.0097; R^2: 0.999, weight: 1/X^1 |
| 59_Glycodeoxycholic Acid-3-Sulfate | 16.25 | Y = -1.79e-06X^2 + 0.0048X - 0.0036; R^2: 0.999, weight: 1/X^1 |
| 60_Glycocholic Acid-3-Sulfate | 6.99 | Y = -5.59e-07X^2 + 0.0056X + 0.0339; R^2: 1.000, weight: 1/X^1 |
| 61_Taurolithocholic Acid-3-Sulfate | 18.89 | Y = -9.00e-07X^2 + 0.0021X - 0.0022; R^2: 0.999, weight: 1/X^1 |
| 62_Chenodeoxycholic acid-3-β-D-Glucuronide | 14.96 | Y = -5.21e-07X^2 + 0.0041X + 0.0176; R^2: 0.999, weight: 1/X^1 |
| 63_Chenodeoxycholic acid 24-Acyl-β-D-glucuronide | 19.87 | Y = -7.04e-06X^2 + 0.0050X + 0.0001; R^2: 0.999, weight: 1/X^1 |
| 64_Tauroursodeoxycholic Acid-3-Sulfate | 5.88 | Y = -2.21e-07X^2 + 0.0013X + 0.0017; R^2: 1.000, weight: 1/X^1 |
| 65_Taurochenodeoxycholic Acid-3-Sulfate | 16.75 | Y = -3.36e-07X^2 + 0.0019X - 0.0021; R^2: 0.999, weight: 1/X^1 |
| 66_Taurodeoxycholic Acid-3-Sulfate | 17.49 | Y = -5.62e-07X^2 + 0.0036X + 0.0030; R^2: 0.999, weight: 1/X^1 |
| 67_Taurocholic Acid-3-Sulfate | 9.28 | Y = -1.37e-06X^2 + 0.0019X - 0.0015; R^2: 0.999, weight: 1/X^1 |
| 68_Glycodeoxycholic acid-3-O-β-glucuronide | 9.98 | Y = 0.0034X + 0.0021; R^2: 0.998, weight: 1/X^1 |
| 69_Glycochenodeoxycholic Acid-3-O-β-glucuronide | 10.33 | Y = 0.0050X + 0.0011; R^2: 0.995, weight: 1/X^1 |

Calibration：

| **Number** | **Name** | **CAS** | **LLOD（nmol/L）** | **LLOQ（nmol/L）** | **ULOQ（nmol/L）** | **R^2** |
| --- | --- | --- | --- | --- | --- | --- |
| 1 | Dehydrolithocholic acid | 1553-56-6 | 0.23 | 0.46 | 121.19 | 0.9982 |
| 2 | Isoallolithocholic acid/Isolithocholic acid | 2276-93-9/1534-35-6 | 0.39 | 0.79 | 238.77 | 0.9971 |
| 3 | Lithocholic acid | 434-13-9 | 0.44 | 0.87 | 121.02 | 0.9972 |
| 4 | 23-Nordeoxycholic acid | 53608-86-9 | 0.22 | 0.44 | 125.79 | 0.9996 |
| 5 | 6-Ketolithocholic acid | 2393-61-5 | 0.81 | 1.62 | 239.70 | 0.9971 |
| 6 | 7-Ketolithocholic acid | 4651-67-6 | 0.46 | 0.91 | 121.30 | 0.9979 |
| 7 | 12-Ketolithocholic acid | 5130-29-0 | 0.22 | 0.45 | 60.37 | 0.9981 |
| 8 | Apocholic acid | 641-81-6 | 6.27 | 12.54 | 969.27 | 0.9978 |
| 9 | Isoursodeoxycholic acid | 78919-26-3 | 0.20 | 0.39 | 251.10 | 0.9995 |
| 10 | Murideoxycholic acid | 668-49-5 | 3.27 | 6.55 | 2013.24 | 0.9996 |
| 11 | Isohyodeoxycholic acid | 570-84-3 | 0.45 | 0.91 | 121.29 | 0.9978 |
| 12 | Ursodeoxycholic acid | 128-13-2 | 0.50 | 1.01 | 122.54 | 0.9988 |
| 13 | Hyodeoxycholic acid | 83-49-8 | 0.72 | 1.45 | 116.97 | 0.9986 |
| 14 | 3-Epideoxycholic acid | 570-63-8 | 3.32 | 6.64 | 2009.50 | 0.9995 |
| 15 | Chenodeoxycholic acid | 474-25-9 | 13.67 | 27.34 | 969.94 | 0.9980 |
| 16 | Deoxycholic acid | 83-44-3 | 13.68 | 27.36 | 972.25 | 0.9980 |
| 17 | Isodeoxycholic acid | 566-17-6 | 0.42 | 0.84 | 121.74 | 0.9984 |
| 18 | Nor Cholic Acid | 60696-62-0 | 1.61 | 3.21 | 2020.16 | 0.9994 |
| 19 | Dehydrocholic acid | 81-23-2 | 0.23 | 0.45 | 118.31 | 0.9966 |
| 20 | 7,12-Diketolithocholic acid | 517-33-9 | 0.40 | 0.80 | 502.66 | 0.9997 |
| 21 | 6,7-Diketolithocholic acid | — | 0.81 | 1.61 | 241.68 | 0.9980 |
| 22 | 7-Ketodeoxycholic acid | 911-40-0 | 3.37 | 6.73 | 242.36 | 0.9976 |
| 23 | 12-Dehydrocholic acid | 2458-08-4 | 0.13 | 0.26 | 121.11 | 0.9977 |
| 24 | 3-Dehydrocholic acid | 2304-89-4 | 0.41 | 0.81 | 121.41 | 0.9976 |
| 25 | Ursocholic acid | 2955-27-3 | 0.43 | 0.86 | 118.94 | 0.9968 |
| 26 | 3β-Cholic Acid | 3338-16-7 | 0.41 | 0.81 | 121.73 | 0.9987 |
| 27 | ω-Muricholic Acid | 6830-03-1 | 13.64 | 27.29 | 968.98 | 0.9979 |
| 28 | α-Muricholic acid | 2393-58-0 | 0.49 | 0.99 | 122.31 | 0.9986 |
| 29 | β-Muricholic acid | 2393-59-1 | 0.44 | 0.88 | 121.67 | 0.9987 |
| 30 | Hyocholic acid | 547-75-1 | 1.65 | 3.31 | 2012.30 | 0.9995 |
| 31 | Allocholic acid | 2464-18-8 | 6.46 | 12.93 | 977.91 | 0.9984 |
| 32 | Cholic acid | 81-25-4 | 6.29 | 12.58 | 952.33 | 0.9964 |
| 33 | Glycolithocholic acid | 474-74-8 | 0.41 | 0.83 | 119.87 | 0.9974 |
| 34 | Glycoursodeoxycholic acid | 64480-66-6 | 0.41 | 0.83 | 503.45 | 0.9996 |
| 35 | Glycohyodeoxycholic acid | 13042-33-6 | 6.44 | 12.88 | 4055.78 | 0.9991 |
| 36 | Glycochenodeoxycholic acid | 640-79-9 | 0.47 | 0.94 | 121.11 | 0.9983 |
| 37 | Glycodeoxycholic acid | 360-65-6 | 0.39 | 0.78 | 119.45 | 0.9957 |
| 38 | Lithocholic Acid-3-Sulfate | 64936-81-8 | 0.41 | 0.82 | 502.59 | 0.9996 |
| 39 | Glycodehydrocholic acid | 3415-45-0 | 0.41 | 0.83 | 118.94 | 0.9967 |
| 40 | Glycohyocholic acid | 32747-08-3 | 0.88 | 1.77 | 504.13 | 0.9995 |
| 41 | Glycocholic acid | 475-31-0 | 0.10 | 0.20 | 30.34 | 0.9984 |
| 42 | Ursodeoxycholic acid 3-Sulfate | 68780-73-4 | 0.40 | 0.81 | 504.30 | 0.9994 |
| 43 | Chenodeoxycholic Acid-3-Sulfate | 59132-32-0 | 0.41 | 0.82 | 119.54 | 0.9965 |
| 44 | Deoxycholic Acid-3-Sulfate | 67030-48-2 | 0.42 | 0.85 | 120.44 | 0.9972 |
| 45 | Taurolithocholic acid | 6042-32-6 | 0.43 | 0.86 | 120.53 | 0.9977 |
| 46 | Cholic Acid-3-Sulfate |  | 0.45 | 0.91 | 119.85 | 0.9975 |
| 47 | Tauroursodeoxycholic acid | 14605-22-2 | 0.44 | 0.87 | 118.42 | 0.9959 |
| 48 | Taurohyodeoxycholic acid | 2958-04-5 | 0.39 | 0.78 | 505.30 | 0.9991 |
| 49 | Taurochenodeoxycholic acid | 516-35-8 | 3.15 | 6.30 | 2012.39 | 0.9995 |
| 50 | Taurodeoxycholic acid | 516-50-7 | 0.90 | 1.79 | 502.96 | 0.9995 |
| 51 | Glycolithocholic Acid-3-Sulfate | 15324-64-8 | 0.45 | 0.89 | 120.06 | 0.9970 |
| 52 | Tauro ω-muricholic acid | 130325-58-5 | 0.40 | 0.79 | 120.18 | 0.9977 |
| 53 | Tauro α-Muricholic acid | 25613-05-2 | 0.41 | 0.82 | 121.16 | 0.9979 |
| 54 | Tauro β-Muricholic acid | 25696-60-0 | 1.57 | 3.14 | 489.62 | 0.9987 |
| 55 | Taurohyocholic acid | 32747-07-2 | 0.10 | 0.20 | 60.19 | 0.9974 |
| 56 | Taurocholic acid | 81-24-3 | 1.63 | 3.25 | 2009.30 | 0.9995 |
| 57 | Glycoursodeoxycholic Acid-3-Sulfate | 133429-88-6 | 0.39 | 0.79 | 119.17 | 0.9965 |
| 58 | Glycochenodeoxycholic Acid 3-Sulfate | 66874-09-7 | 3.42 | 6.83 | 2018.28 | 0.9993 |
| 59 | Glycodeoxycholic Acid-3-Sulfate | 66874-10-0 | 0.83 | 1.66 | 504.56 | 0.9992 |
| 60 | Glycocholic Acid-3-Sulfate | 67850-84-4 | 3.33 | 6.65 | 2010.08 | 0.9997 |
| 61 | Taurolithocholic Acid-3-Sulfate | 15324-65-9 | 0.52 | 1.05 | 502.11 | 0.9991 |
| 62 | Chenodeoxycholic acid-3-β-D-Glucuronide | 58814-71-4 | 3.17 | 6.34 | 2025.73 | 0.9990 |
| 63 | Chenodeoxycholic acid 24-Acyl-β-D-glucuronide | 208038-27-1 | 0.22 | 0.43 | 125.88 | 0.9994 |
| 64 | Tauroursodeoxycholic Acid-3-Sulfate |  | 1.67 | 3.34 | 1006.75 | 0.9995 |
| 65 | Taurochenodeoxycholic Acid-3-Sulfate | 67030-59-5 | 3.32 | 6.64 | 1007.50 | 0.9994 |
| 66 | Taurodeoxycholic Acid-3-Sulfate |  | 1.73 | 3.45 | 1014.41 | 0.9987 |
| 67 | Taurocholic Acid-3-Sulfate | 67030-62-0 | 0.48 | 0.95 | 252.09 | 0.9993 |
| 68 | Glycodeoxycholic acid-3-O-β-glucuronide | 75672-36-5 | 0.40 | 0.81 | 123.05 | 0.9982 |
| 69 | Glycochenodeoxycholic Acid-3-O-β-glucuronide | 75672-22-9 | 1.59 | 3.18 | 247.21 | 0.9960 |
